# Supplementary material for: MEG cortical microstates: Spatiotemporal characteristics, dynamic functional connectivity and stimulus-evoked responses
Source: Neuroimage. 2022 May 1;251:119006. doi: 10.1016/j.neuroimage.2022.119006 (PMC8961001; doi:10.1016/j.neuroimage.2022.119006)
Supplement: Supplementary Data S1 — Supplementary Raw Research Data. This is open data under the CC BY license http://creativecommons.org/licenses/by/4.0/ [file mmc1.pdf]

Supplementary Material for "MEG cortical microstates: spatiotemporal characteristics, dynamic functional connectivity and stimulus-evoked responses"

Luke Tait[1,2] and Jiaxiang Zhang[2]

[1]Centre for Systems Modelling & Quantitative Biomedicine (SMQB), University of Birmingham, Birmingham, UK
[2]Cardiff University Brain Research Imaging Centre, Cardiff, UK

S1 Supplementary Methods

S1.1 Mathematical description of the generalized microstate-pipeline

Let $X \in \mathbb{R}^{N\times T}$ be an electrophysiological data set, where N is the spatial dimensionality (number of M/EEG sensors, or source space dipoles/ROIs) and T is the number of time samples. Each column of X is a spatial map, i.e. a sample of data $\mathbf{x}(t) \in \mathbb{R}^{N\times 1}$. Then the aim of microstate analysis is to identify a set of spatial maps with high signal-to-noise ratio (SNR) and cluster these maps into a small number of discrete states (Pascual-Marqui et al., 1995; Koenig et al., 1999; Khanna et al., 2015; Michel and Koenig, 2018). The most widely used methodology for EEG is the modified k-means algorithm presented by Pascual-Marqui et al. (1995) and Koenig et al. (1999), although a range of methods exist (Michel and Koenig, 2018) which have been demonstrated to give consistent spatial topographies (Khanna et al., 2014) and information-theoretically invariant temporal sequences (von Wegner et al., 2018). There are steps in the EEG microstate methodology which limit application to MEG sensor space or M/EEG source space data (outlined in Main Text Section 2.1), and to overcome this we here present the EEG microstate k-means algorithm (Pascual-Marqui et al., 1995; Koenig et al., 1999) in a framework which allows for generalization to other large scale electrophysiological datasets such as M/EEG sensor and source space based on an appropriate modality-specific transformation of the each spatial map $\mathbf{y} = f(\mathbf{x})$.

In this generalized framework, the EEG algorithm (Pascual-Marqui et al., 1995; Koenig et al., 1999) is recovered by re-referencing the data to a common average, i.e. using the transformation $\mathbf{y} = \mathbf{x} - \mu_{\mathbf{x}}$, where $\mu_{\mathbf{x}}$ is the average of $\mathbf{x}$ over sensors. The equivalent transformations for other modalities are given in Table S1. For sensor MEG, no transformation of the data is required (including no re-referencing). For source space, where source flipping may be an issue, there are two options. One is to use the amplitude envelope of the data (row labelled "Amplitude" in Table S1). However, this requires a narrow band signal and rejects phase information. A second option is to simply take the (element-wise) absolute value of the samples (row labelled "Source" in Table S1), which can be applied to broadband data and mostly maintains phase information (excluding phase differences of π).

The first step of the pipeline involves extracting samples with local peaks of SNR and map stability. These samples are peaks of the *global field power* (GFP), defined at a given time within our generalized framework as

$$\sigma(t) = \sqrt{\frac{1}{N-1}}\|\mathbf{y}(t)\| = \sqrt{\frac{1}{N-1}\sum_{n=1}^{N} y_n(t)^2} \qquad (1)$$

The GFP is therefore the total deviation of the transformed map $\mathbf{y}$ from zero across space. For EEG, in which $y_n = x_n - \mu_{\mathbf{x}}$, this is equal to the standard deviation of the map, i.e. the total variation from the common average reference. In MEG and source space, the function is more appropriately viewed as a normalized vector norm of the map, and is therefore the total variation from zero field/current. Peaks of the GFP were here found using

Modality	Spatial transformation
EEG	$\mathbf{y} = \mathbf{x} - \mu_{\mathbf{x}}$
MEG	$\mathbf{y} = \mathbf{x}$
Source	$\mathbf{y} = (\lvert x_1\rvert, \lvert x_2\rvert, \ldots, \lvert x_N\rvert)^T$
Amplitude	$\mathbf{y} = (\lvert H(x_1)\rvert, \lvert H(x_2)\rvert, \ldots, \lvert H(x_3)\rvert)^T$

Supplementary Table S1: **Transformations used in generalized formulation of clustering analysis for each modality**. Here, $\mathbf{x} \in \mathbb{R}^{N\times 1}$ is a spatial topography with spatial dimension N (i.e. a single time sample of multivariate data) with elements $(\mathbf{x})_n = x_n$, $\mu_{\mathbf{x}}$ is the mean of $\mathbf{x}$, $|\cdot|$ denotes absolute value, and $H(\cdot)$ denotes the (temporal) Hilbert transform (therefore $|H(\cdot)|$ is the instantaneous amplitude envelope).

Matlab's findpeaks function. Appropriate pre-processing of the data including bandpass filtering is important here to generate a smooth GFP signal for accurate identification of peaks.

Once the set of GFP peaks has been extracted, we cluster the set of transformed spatial maps at these peaks into k clusters. The k-means algorithm is outlined in Table 2 of the main text, and a clear graphical representation is given in Koenig et al. (1999) and Michel et al. (2009). The number of states k is a free parameter, and must be chosen appropriately for the data. The algorithm initializes (step #1 in Main Text Table 2) by randomly selecting k transformed maps as initial state centroids $\mathbf{C} \in \mathbb{R}^{N\times k}$. Here, we apply the k-means++ method of selecting initial maps (Arthur and Vassilvitskii, 2007; Tait et al., 2020), which has better performance and faster convergence than uniform sampling (Arthur and Vassilvitskii, 2007) which has commonly been used for microstate studies (Pascual-Marqui et al., 1995; Koenig et al., 1999; Michel and Koenig, 2018).

The subsequent steps of the algorithm are iterated until some convergence critera are met. Firstly, the distance between each transformed map and each cluster centroid is calculated (step #2 in Main Text Table 2). It is convenient to first define the *similarity* between a (transformed) map $\mathbf{y}$ and a centroid $\mathbf{c}$ (i.e. a column of $\mathbf{C}$) as

$$R(\mathbf{y}, \mathbf{c}) = \frac{|\mathbf{y}^T \cdot \mathbf{c}|}{\sqrt{\mathbf{y}^T \cdot \mathbf{y}}\sqrt{\mathbf{c}^T \cdot \mathbf{c}}}. \tag{2}$$

This equation is often called the *cosine similarity*, as it can equivalently be written as

$$R(\mathbf{y}, \mathbf{c}) = |\cos(\theta(\mathbf{y}, \mathbf{c}))|, \tag{3}$$

where $\theta(\mathbf{y}, \mathbf{c})$ is the angle between the two vectors $\mathbf{y}$ and $\mathbf{c}$, and hence it is clear that this value is in the range zero (orthogonal maps) to one (maps point in the exact same direction, ignoring polarity).

Subsequently, we can define the *distance* between maps as

$$D(\mathbf{y}, \mathbf{c}) = 1 - R(\mathbf{y}, \mathbf{c}) \tag{4}$$

For EEG, where both $\mathbf{y}$ and $\mathbf{c}$ have zero mean, it is clear from Equation 2 that the similarity is the absolute value of correlation, in line with the classic microstate k-means clustering algorithm (Pascual-Marqui et al., 1995; Koenig et al., 1999). For MEG and source data this can more readily be interpreted as the cosine similarity, i.e. the absolute value of the cosine of the angles between the vectors $\mathbf{y}$ and $\mathbf{c}$. For all modalities, $0 \le D \le 1$, where a value of one represents uncorrelated/orthogonal spatial maps, while a value of zero represents identical maps up to a scaling factor, i.e. $D(\mathbf{y}, \mathbf{c}) = 0 \iff \mathbf{y} = \alpha\mathbf{c}$, where $\alpha \in \{\mathbb{R} \setminus 0\}$.

Each map is subsequently labelled as belonging to the cluster with which it has minimal distance (or maximal similarity) to the cluster centroid (step #3 in Main Text Table 2), i.e.

$$L_i = \min_j(D(\mathbf{y}_i, \mathbf{c}_j)) \equiv \max_j(R(\mathbf{y}_i, \mathbf{c}_j)), \tag{5}$$

where subscript i represents the i'th GFP peak with map $\mathbf{y}_i$, j represents the j'th cluster with centroid $\mathbf{c}_j$, and $L_i \in \{1, 2, \ldots, k\}$ is the label assigned to peak i.

The centroid for each cluster is then updated (step #4 in Main Text Table 2). For a given cluster j containing M maps, let us define the matrix $\mathbf{Y}_j \in \mathbb{R}^{N\times M}$ such that each column of $\mathbf{S}_j$ is one of the transformed maps $\mathbf{y}$ in the cluster. As in the algorithm of Pascual-Marqui et al. (1995), the updated centroid of cluster j is here defined

as the eigenvector of $\mathbf{Y}_j^T\mathbf{Y}_j$ corresponding to the largest eigenvalue. While not strictly necessary for the k-means algorithm described above, it is typical to normalize the resulting centroids to unit vector norm (Pascual-Marqui et al., 1995), a process which is performed automatically in many implementations of eigenvalue decomposition, including the implementation used here (Matlab's eig function).

With the updated cluster centroids, the algorithm returns to step #2 (Main Text Table 2), iteratively recalculating distances between maps and centroids, relabelling states, and recalculating the centroids. These steps are iterated until convergence is met, i.e. once there is no change in assigned cluster labels between subsequent iterations (Koenig et al., 1999), or up to a fixed maximum number of iterations, here chosen to be 100 iterations (Tait et al., 2020). Since the choice of initial maps is random, different runs of the algorithm may give different results on the same dataset. For this reason, we repeat the clustering 20 times using a different initial seed on each repeat (Koenig et al., 1999; Tait et al., 2020). The repetition with the highest globally explained variance (Murray et al., 2008, GEV) is used in subsequent analysis, where GEV is defined as:

$$\text{GEV} = \frac{\sum_i \sigma_i^2 \max_j (R^2(\mathbf{y}_i, \mathbf{c}_j))}{\sum_i \sigma_i^2}, \tag{6}$$

where $\sum_i$ denotes summation over samples, σ_i is the GFP at sample i (Equation 1), and $R(\mathbf{y}_i, \mathbf{c}_j)$ is the similarity between the map at sample i and the centroid of cluster j (Equation 2). It should be noted here that the phrase GEV is maintained in this study to align with microstate literature, but in reality this measure is only a true measure of explained variance (by a linear model) when R^2 is squared correlation, i.e. the case of sensor EEG signals. However, interpretation is highly similar for all modalities. GEV is always in the range $[0, 1]$, and when GEV=1, the cluster centroid maps can fully explain the data, while GEV=0 indicates the maps do not explain any data.

In the EEG microstate literature, a range of methods exist for clustering states (Michel and Koenig, 2018). The most widely used alternative to k-means is topographic atomize and agglomerate heirarchical clustering (TAAHC). TAAHC has been demonstrated to give consistent spatial topographies (Khanna et al., 2014) and information-theoretically invariant temporal sequences (von Wegner et al., 2018) when compared to k-means in EEG microstate sequences. TAAHC is notably more computationally expensive than k-means for large datasets (von Wegner et al., 2018), motivating our choice for k-means in this study. However, the TAAHC algorithm suffers from the same technical limitations when applied to source/MEG data as the original k-means formalism, and therefore the spatial transformations and generalized definitions of GFP and map (dis)similarity presented here may also be used to adapt the TAAHC algorithm for source/MEG microstates.

S1.2 Optimum number of microstates

A number of methods exist for choosing the optimum number of clusters/microstates from the data (Michel and Koenig, 2018). The most widely used in the EEG microstate literature are the Cross Validation Index (CVI) (Pascual-Marqui et al., 1995) and the Krzanowski-Lai (KL) criterion (Murray et al., 2008). CVI is highly dependent on number of sensors/ROIs, and is known to perform poorly for large numbers of ROIs (≥ 64 according to Murray et al. (2008)). Here, we used 274 channel MEG mapped to 230 source space ROIs, so we did not use CVI. While KL criterion has been used for resting-state microstate analysis (Hatz et al., 2015; Tait et al., 2020), it is believed to work appropriately for event-related potential analysis but result in spurious peaks for resting-state data Michel and Koenig (2018). Alternatively, Custo et al. (2017) considered eleven different criteria for the optimum number of microstates and developed a meta-criterion combining these solutions.

Here, we use the kneedle algorithm (Satopää et al., 2011) to choose the number of states. As the number of clusters k increases, so does the variance explained. Increasing k up to the true number of states will only give a large increase in GEV as more states of the brain are accounted for, whereas increasing k above the true number of states only explains noise and hence will give marginal increases to GEV. Therefore the plot of GEV vs k has a characteristic 'knee' shape. The kneedle algorithm aims to find the number of clusters in the dataset by finding the value of k for which this knee occurs. This criterion is simple, intuitive, and easy to interpret. It does not depend on number of channels, and does not generate multiple spurious peaks due to the smooth increase of GEV with number of clusters. For this reason, we here used kneedle for the optimum number of states.

S1.3 Simulations

Simulations were performed to test the ability of the microstate methodology to estimate a ground truth microstate sequence and spatial maps. Simulations were repeated for $k = 4$ and $k = 10$ states. The three main steps to simulations are described below.

Firstly, a generative microstate sequence was simulated. While any sequence of numbers could be used here, we aimed to generate a sequence with EEG-microstate like properties and avoid assumptions known to be invalid for EEG microstates such as Markovianity or stationarity. To do so, we used a random walk decision tree approach, which reverse engineers the method commonly used to transform EEG microstate sequences to random walks using a decision tree (Van De Ville et al., 2010; von Wegner et al., 2016, 2018). $k - 1$ random walks were generated with Hurst exponent 0.7 (Van De Ville et al., 2010). Each random walk was then converted to a binary sequence based on the sign of their derivative, i.e. +1 if they are increasing, and -1 for decreasing. Each binary sequence corresponds to a branch of a decision tree, which places a particular time point within a state. For the example of four states, the first split separates the states {1,2} from states {3,4}, while the second split separates states 1 and 2, and the third split separates states 3 and 4. For example, consider a time point with the three binary sequences having values of (+1,-1,+1). The first binary sequence is +1, so the current state is either state 1 or 2 (a value of -1 would indicate state 3 or 4). To decide which of these two states is the current sample, we consider the 2nd sequence (which separates states 1 and 2; if the first sequence had a value of -1 we could consider the 3rd sequence which separates states 3 and 4). Values of +1/-1 in the 2nd sequence correspond to states 1 and 2 respectively, so since the 2nd sequence had a value of -1 the state at this time point is 2. Prior to binarizing the states, we smoothed the random walk with a moving average filter. The width of the filter was chosen for each simulation such that the mean duration was approximately 50 ms (Tait et al., 2020). We will denote the resulting sequence L, which is a vector of length T (the number of samples), where the t'th sample $L(t) \in \{1, 2, \ldots, k\}$. This method resulted in generative sequences with mean duration of approximately 50 ms, Hurst exponents of approximately 0.7-0.8, and which were highly non-Markovian in line with past EEG microstate studies (Van De Ville et al., 2010; von Wegner et al., 2017, 2018; Tait et al., 2020).

Neural dynamics were then generated for each microstate. Each state was assigned a Wilson-Cowan neural mass model in a parameter regime previously used to model resting-state dynamics (Deco et al., 2009; Abeysuriya et al., 2018). For state j, the equations of the model were

$$\tau_E \dot{E}_j = -E_j + \phi(w_+ E_j - I_j + P_0 + P_{ms}(t)) + \sigma \nu_E(t) \quad (7)$$

$$\tau_I \dot{I}_j = -I_j + \phi(w_I E_j) + \sigma \nu_I(t) \quad (8)$$

$$P_{ms}(t) = \begin{cases} P_1 & \text{if } L(t) = j \\ 0 & \text{otherwise} \end{cases} \quad (9)$$

Here, E_j and I_j correspond to the firing rates of pools of excitatory and inhibitory neurons associated with microstate j respectively. ν_E and ν_I are independent Gaussian noise terms with variance 1. The sigmoid function $\phi(x)$ is given by

$$\phi(x) = \frac{c}{1 + \exp(-a(x - b))}. \quad (10)$$

The remaining parameters and their interpretations are given in Table S2.

The input P_0 was chosen by Deco et al. (2009) such that (in the absence of microstate related drive P_{ms}) the model has a single stable fixed point close to a Hopf bifurcation, and as such exhibits low amplitude aperiodic noise-driven fluctuations with resonance at an alpha band frequency. Therefore all $k - 1$ neural masses with $P_{ms} = 0$ at any given time are in this regime. When microstate associated drive is added ($P_{ms} = P_1$), the system moves across the Hopf bifurcation into a high-amplitude stable periodic alpha oscillation. The LFP of each microstate was given by the sum of synaptic input onto the excitatory population,

$$s_j(t) = w_+ E_j(t) - I_j(t), \quad (11)$$

where s_j is the observed LFP for microstate j.

Simulations were performed using the stochastic Heun algorithm with time step of 0.1ms. Each simulation was initialized with 2 seconds to reach steady states, and then 60 seconds of data was simulated. Simulated

Parameter	Description	Value
τ_E	Excitatory time constant	10 ms
τ_I	Inhibitory time constant	20 ms
w_+	Strength of recurrent excitation	1.4
w_I	Strength of excitation onto inhibitory neurons	1.5
P_0	Background input to excitatory neurons	10
P_1	Microstate associated input to excitatory neurons	3
σ	Standard deviation of neural noise	0.1
c	Maximal firing rate	100
b	Firing threshold	40
a	Slope of sigmoid	0.1

Supplementary Table S2: Parameters of the neural mass model

data was subsequently downsampled to 256 Hz in line with the empirical data.

Finally, the states were assigned spatial maps. As with the choice of generative sequence, the choice of spatial map is arbitrary. Here we used open acess fMRI derived resting-state network maps (Smith et al., 2009) aligned to our 230 ROI atlas by averaging all voxels within an ROI. The result was a matrix $W \in \mathbb{R}^{230\times k}$, whose columns are the spatial maps for each state. If we define $S \in \mathbb{R}^{k\times T}$ to be a matrix containing the neural dynamics of each state, i.e. $S_{j,t} = s_j(t)$, then the simulated LFP at the 230 ROIs were given by $X = WS + \lambda\eta$, where $X \in \mathbb{R}^{230\times T}$ has rows corresponding to time series for each ROI. $\eta \in \mathbb{R}^{230\times T}$ is pink noise, and λ is a scalar factor for the noise chosen such that SNR was 1.

S1.3.1 Comparison with other state-estimation methodologies

Because simulations have ground truth maps and microstate sequences, they were used to compare k-means against other clustering algorithms:

- Principal Component Analysis (PCA), using Matlab's implementation (https://uk.mathworks.com/help/stats/pca.html). PCA is an outer product model in which each component is described by an activation map and a component time courses. In PCA, the first component is the map/time-course pair which maximally explains variance across the dataset. Each subsequent component is temporally uncorrelated to previous components and maximises the remaining variance explained. Component time courses were thresholded in a winner-takes-all approach to obtain discrete state time courses.
- Independent Component Analysis (ICA), using the fastica algorithm (Hyvarinen, 1999). Much like PCA, ICA is an outer product model in which each component is described by an activation map and a component time course. However, while PCA tries to maximise variance explained, ICA aims to unmix true independent sources underlying the signal by maximising non-Gaussianity of component time courses. As in PCA, discrete state activations were achieved using winner-takes-all.
- Hidden Markov Modelling (HMM), using the HMM-MAR toolbox (https://github.com/OHBA-analysis/HMM-MAR). HMMs are a broad framework in which the data is modelled as being generated by a series of hidden states, each of which are associated with an observation model. We used the recommended settings for raw source and amplitude data in the HMM-MAR toolbox, which are based upon (Vidaurre et al., 2018) and (Baker et al., 2014) respectively. For amplitude data, the observation model associated with each state is a Gaussian process generated from a state-specific covariance matrix. For raw source data, the observation model is assumed to be a time-delay embedded covariance matrix, which gives both time-lagged covariance and spectral information for each state.

Four statistics were used to assess the quality of the state estimation. Firstly, GEV was used as a purely data driven quantity. Secondly, we correlated the estimated spatial maps for each state against the ground truth maps using a template matching algorithm. Third, we assessed the temporal sequence accuracy using the normalized mutual information (MI) between the ground truth and estimated sequence. Finally, we also calculated the normalized mutual information only at the GFP peaks (MIg), since these are the samples with locally maximal SNR.

S1.3.2 Phase locking simulations

To demonstrate differences in interpretation between different clustering methods described in the previous section, we additionally ran some simulations for a simple 5 node model consisting of 3 states:

- In state 1, all nodes had a 1/f spectrum with an alpha (10 Hz) peak. All nodes were uncorrelated (initial phases for each frequency and node IID distributed and drawn from $U(0, 2 * pi)$).
- State 2 was identical to state 1, except alpha rhythms were coherent (initial phases for frequencies 8-12 Hz were IID distributed and drawn from $N(0, 0.1)$, introducing phase locking in the alpha frequency band).
- State 3 was identical to state 1, except the peak was at a beta frequency (17 Hz) instead of 10 Hz.

Dynamics for node i in state j were given by:

$$x_i(t) = \sum_f A_j(f) \sin(2\pi f t + \phi_j^0(f)), \quad (12)$$

$$A_j(f) = 1/f + exp(-(f - f_{0,j})^2) \quad (13)$$

where the state-specific peak frequencies ($f_{0,j}$) and initial phases ($\phi_j^0(f)$) are described in the descriptions of each state above. Frequencies were simulated from 1-100 Hz in steps of 0.2 Hz.

We simulated 20 trials, each containing 5 minutes of data sampled at 200 Hz. State durations were uniformly distributed from 20ms to 1s, while transitions were drawn from a Markov chain with no self loops. The elements of the Markov matrix were randomly drawn from a uniform distribution (except the diagonals, which were zero) and each row summing to 1. State estimation via each of the clustering methods (k-means, HMM, PCA, ICA) was performed at the group level by concatenating each trial.

Crucially, these simple simulations aim to highlight some differences between methods. Microstates, PCA, and ICA define states in terms of patterns of instantaneous activation (topographic maps for microstates, spatial vectors for PCA/ICA components). The time-delay embedded HMM conversely defines states in terms of covariance and spectral content. Hence, the states for this simulation are set up in such a manner that microstates, PCA, and ICA are likely to fail (due to all states having equal power across all nodes), but HMMs should succeed in differentiating the states.

S1.4 Microstate segmented functional connectivity

Our method is described below, and a visual overview of the pipeline is given in Figure S1. We first assign each time point in the broadband (1-30 Hz) data a microstate class using the methods described above. We subsequently filter the data into one of three narrow bands (theta 4-8 Hz, alpha 8-13 Hz, beta 13-30 Hz), and calculate the analytical signal using the Hilbert transform. For a given microstate, we concatenate all samples of the analytic signal within this microstate class, and use this to derive time courses of instantaneous phases and amplitudes. While these time series are no longer continuous, the Hilbert transform was performed on the continuous data so they represent instantaneous phases and amplitudes of the continuous signal at these samples. Under the assumption that microstates are associated with specific patterns of phase synchronization (i.e. the hypothesis we wish to test), we can subsequently calculate functional connectivity from the concatenated phase/amplitude time courses. A large number of metrics exist to quantify functional connectivity. Since amplitude coupling is typically performed on a slower time scale through lowpass filtering and downsampling, here we chose to address microstate synchronization using phase coupling, specifically the weighted phase lag index (wPLI) (Vinck et al., 2011; Colclough et al., 2016), which is a reliable estimator of phase coupling (Colclough et al., 2016) while attenuating spurious connections due to leakage Vinck et al. (2011). The (unweighted) phase lag index was used by Hatz et al. (2015) in their study of microstate connectivity, but wPLI is more reliable and less biased due to small noisy perturbations than PLI (Vinck et al., 2011; Colclough et al., 2016). This method is repeated for each microstate class, to obtain functional connectivity patterns for each class.

The methodology described above assumes that microstates are associated with a unique pattern of phase synchronization, and therefore by concatenating samples within a particular class we obtain a stationary time course from which it is meaningful to calculate a single functional network. To test this hypothesis, we applied multi-variate pattern analysis (MVPA) using the MVPA-Light toolbox (Treder, 2020). For each frequency band,

the following analysis was performed. Firstly, for each participant, scan, and microstate class, we followed the methodology above for calculating microstate functional connectivity. However, prior to calculation of wPLI, the concatenated phase/amplitude time-courses were segmented into non-overlapping windows of 1280 samples (equivalent to 5-seconds). Across all participants, scans, and microstate classes this resulted in 5413 networks per frequency. Each network was associated with a single microstate class. For each network, the weighted degree distribution (i.e. sum of all edges connecting to a node) was calculated, resulting in a 230 (number of ROIs) dimensional vector for each network. These 230 dimensional vectors were used as features in a multi-class linear discriminant analysis classifier, with the associated microstate class as the class-labels to be predicted. Classification used default values in the MVPA-Light toolbox, while 5-fold cross-validated classification accuracy was used as the metric of performance. If high classification accuracy is achieved, this is strongly suggestive that microstates are associated with unique patterns of phase synchronization, since it suggests that given any 1280 samples from a given microstate class (across participants/scans), this class can be predicted based on the functional connectivity pattern. Permutation testing was used to test for statistically significant classification accuracy, using 1000 permutations.

Finally, for visualization purposes only, we plotted the deviation of the microstate functional connectivity pattern from the static connectivity pattern. This was done since plotting raw microstate functional connectivity patterns gave visually similar results, suggesting an underlying static connectivity associated with all states. Firstly, we obtained a single microstate network (for each class) by averaging the network across all 5-second segments within the class, participants, and scans. We obtained a single static network (used for all classes) by segmenting the original data into 5-second epochs, calculating wPLI, and averaging the resulting networks over all epochs, participants, and scans. We subsequently normalized the microstate-connectivity by the static connectivity using the equation

$$\text{wPLI}^{ij}_{\text{deviation}} = \frac{\text{wPLI}^{ij}_{\text{microstate}} - \text{wPLI}^{ij}_{\text{static}}}{1 - \text{wPLI}^{ij}_{\text{static}}}, \tag{14}$$

where superscripts i, j denote ROIs i and j. This equation has in the past been used to remove linear-connectivity from non-linear connectivity matrices (Rummel et al., 2015), but here is applied to remove static connectivity from microstate-connectivity matrices. For visualization, the top 1% of edges from the matrix $\text{wPLI}_{\text{deviation}}$ are shown.

S2 Supplementary Results

S2.1 Reproducibility and reliability of maps

To test the reliability and reproducibility of the estimated microstate maps, we repeated the k-means clustering 250 times using a different random seem and sampling a different selection of 150,000 GFP peaks. For each of these permutations, only a single repetition of the algorithm was performed (unlike the original run, where 20 repetitions were used and the repetition with highest GEV was chosen). The estimated 10 maps on each of these iterations were aligned to the original 10 maps using iterative template matching, i.e. map similarity between the estimated maps and the templates (the original 10 maps) was calculated according to Equation 2, and the template/map pair with highest similarity were given the same label. Then both this template and map were removed from the pool. This was repeated until all maps were aligned.

To quantify how tightly the 250 runs were clustered about the original maps, we use the Silhouette coefficient (Rousseeuw, 1987). The silhouette coefficient has a value for each sample (i.e. 250 repetitions and 10 clusters), ranging from -1 (poorly clustered) to +1 (well clustered). Distance between maps was calculated using Equation 4. The measure rewards low intra-cluster distances and high inter-cluster distances. Therefore if the estimated maps for all 250 runs are highly repeatable, the template matching algorithm will place each run in approximately the same location in the abstract space spanned by the map values, resulting in low intra-cluster distances (and high inter-cluster distances relative to the low intra-cluster distances) and therefore large silhouette coefficient.

Results are shown in Figure S2. On average, there was high similarity between our microstate maps and maps estimated from subsequent runs, and the mean ($\pm$ standard error) of similarity values across all classes and runs was 0.9668 ± 0.0019. The silhouette coefficient was 0.7726 ± 0.0047, indicating that the various repetitions were well clustered. Of the maps, the frontal and left/right parietal were the least reproducible (still having

Statistic	Value	p-stationary
GEV	$63.05 \pm 0.41\%$	3.1×10^{-16}
MD	59.86 ± 1.09 ms	1.6×10^{-7}
H	0.6829 ± 0.0103	1.3×10^{-3}
C	0.5986 ± 0.0134	1.9×10^{-11}
stdCov	$14.28 \pm 0.59\%$	0.5667
stdDur	15.89 ± 1.03 ms	5.8×10^{-5}
stdOcc	1.913 ± 0.059 s^{-1}	2.3×10^{-7}
**Statistic**	**Value**	**Markov 95%**
G_0	$2.881 \pm 0.091 \times 10^5$	103.0
G_1	$1.242 \pm 0.049 \times 10^3$	877.3

Supplementary Table S3: Statistics of MEG microstates

silhouette coefficients > 0.4 and map similarities > 0.83, indicating high reliability).

S2.2 Assessing non-stationarity

An important point to note is that the k-means clustering algorithm will cluster the data even in the absence of true stable microstates. We therefore additionally tested whether the statistics of the microstate sequences could be replicated by random fluctuations in a stationary Gaussian process, or whether they required genuine non-stationarity and the appearance of stable states. To do so, for each participant and scan, we generated 100 multivariate iterative amplitude adjusted Fourier transform (mvIAAFT) surrogates (Schreiber and Schmitz, 2000). These surrogate time series are stationary Gaussian processes with the same power/cross spectra and distributions as the original dataset (Schreiber and Schmitz, 2000; Rummel et al., 2011). Therefore if the microstate statistics from the empirical MEG data were drawn from the same distribution as the microstate statistics from the surrogate data, it is probable that the observed 'microstates' are really just due to random fluctuations of a stationary process. Similar approaches have been taken to statistically analyse non-stationarity in dynamic functional connectivity in neuroimaging datasets (O'Neill et al., 2018). Here, we found no surrogate distributions were significantly different from a normal distribution (minimum p-value over all participants, scans, and microstate statistics was $p = 0.2310$ using a Kolmogorov-Smirnov test), so therefore quantified differences in the empirical statistics vs the surrogates via a z-score, i.e. $z = (x - \mu_{\text{surrogate}})/\sigma_{\text{surrogate}}$, where x is the empirical statistic of interest, and $\mu_{\text{surrogate}}$ and $\sigma_{\text{surrogate}}$ are the mean and standard deviation of the surrogate distributions respectively. For each statistic, the z-scores were subsequently averaged over scans, giving a single z-value per participant. To test whether the resulting distribution of z-scores across participants was likely drawn from a normal distribution with mean zero (i.e. whether the z-scores were significantly different from the surrogates), a one sample t-test was used for each statistic. For this analysis, the statistics which were class-specific (duration, coverage, occurrence) were transformed to a standard deviation across classes, so the test estimated specifically whether heterogeneity between microstates in these properties could be accounted for in a stochastic process.

Results of the non-stationarity analysis are shown in Supplementary Table S3 and Supplementary Figure S3. Mean duration of microstates was significantly larger than could be explained by random fluctuations in a stationary Gaussian process with the same power spectra, cross-spectra (and covariances), and distribution of data, suggestive of non-stationarity and the occurence of stable microstates in empirical resting-state MEG. Heterogeneity in durations across states (quantified by standard deviation; stdDur) was also significantly larger in empirical data than due to chance.

S2.3 Comparing state-estimation methods based on simulations

In this section, we aim to compare and contrast the different methods of brain-state estimation outlined in section S1.3.1 via simulations. Two models were used, outlined in Supplementary Table S4.

The 'activation' model (section S1.3, main text Figure 1) is set up in such a way that meets the assumptions of k-means, PCA, and ICA. States are defined in terms of spatial maps, i.e. each state has a unique pattern of activations. While the ground-truth state sequences were discrete, i.e. only one state was active at a given

Model	Trial length (mins)	N_{nodes}	Analysis-level	Transitions	State definition
Activation	1	230	Trial	Random-walk	Spatial activation pattern
Phase-locking	5	5	Group	Markov chain	Peak frequency & phase-locking

Supplementary Table S4: Summary of models used for comparing state-estimation methods

	**Act/source/4**	**Act/amp/4**	**Act/source/10**	**Act/amp/10**	**Phase**
k-means	40.3 (38.4-41.0)	27.8 (26.5-28.5)	45.0 (44.1-46.2)	35.1 (34.0-36.0)	1.4 (1.3-1.6)
PCA	45.4 (43.9-47.2)	35.0 (34.1-36.3)	43.9 (41.7-46.4)	37.0 (33.9-40.0)	2.7 (2.6-2.8)
ICA	31.7 (30.2-33.7)	27.2 (25.1-29.3)	37.6 (35.7-40.4)	33.6 (32.2-37.4)	1.6 (1.4-1.8)
HMM	1.0 (0.7-1.2)	0.8 (0.4-1.3)	3.7 (3.2-3.9)	4.2 (3.8-4.3)	47.2 (46.9-47.9)

Supplementary Table S5: Mutual information between ground-truth and estimated state sequences in the various simulation scenarios. Values are given as a percentage, and shown are median and interquartile range (in brackets). Abbreviations: Act/source/k is the activation model applied to raw time courses with k simulated states. Act/amp/k is the activation model applied to amplitude envelopes with k states. Phase represents the phase-locking model.

time, some degree of mixing between states was allowed since even inactive states exhibited low-amplitude noise-driven resonant oscillations, while active states exhibited high-amplitude limit-cycle oscillations. These simulations were not set up well for HMMs, since states were not defined in terms of covariance matrix or spectral content. Furthermore, the non-Markovian nature of the state sequences may be a limiting factor for the performance of HMMs (Koenig and Brandeis, 2016). In addition, the complexity of HMMs will greatly increase with dimensionality, and hence for a 230 node system may require a large amount of data to train (e.g. Baker et al. (2014) used 9 subjects $\times$ 10 minutes of data with 40 nodes, and Vidaurre et al. (2018) used 55 subjects $\times$ 5 minutes of data with 42 nodes), while in these simulations clustering was performed separately for each trial.

Results of the activation simulations are shown in Supplementary Table S5. Across both raw and source time courses, as well as 4 and 10 states, HMMs consistently performed worst as expected. MI between simulated and estimated state sequences ranged between 0.8 and 4.2%, depending on model configuration. Conversely k-means, PCA, and ICA had values ranging from 27.2-45.4% across all model configurations. Broadly speaking, PCA and k-means were best performing in terms of both state sequence estimation (Supplementary Table S5) and spatial map estimation (Supplementary Table S6).

The 'phase-locking' model (section S1.3.2) is set up in such a was that meets the assumptions of HMMs, while failing to meet any of the assumptions for k-means, PCA, or ICA. States are defined in terms of phase-locking and frequency peaks, while all states have uniform activation across all nodes. Hence, in theory the methods which define states in terms of activation patterns should fail. To better allow for HMM training, we kept the dimensionality low (5 nodes), simulated 20 trials of 5 minutes in length, and performed analysis on the group-level (i.e. fit states to all trials simultaneously). Results are shown in Supplementary Table S5. k-means, PCA, and ICA all performed poorly (MI between ground-truth and estimated sequences ranging from 1.4-2.6%), while HMMs performed well (MI of 47.2%).

It is important to note that both sets of simulations are likely extreme examples and empirical M/EEG data will to some degree meet assumptions of both models. For example, if the variances of nodes (i.e. diagonals of the covariance matrix) change between states, then this will be reflected in patterns of activation. Spectral changes

	**Act/source/4**	**Act/amp/4**	**Act/source/10**	**Act/amp/10**
k-means	2.449 (2.420-2.485)	1.680 (1.664-1.691)	1.258 (1.218-1.268)	1.302 (1.288-1.331)
PCA	1.951 (1.748-2.069)	1.726 (1.705-1.737)	1.130 (1.003-1.197)	1.043 (1.004-1.074)
ICA	0.647 (0.559-0.750)	0.997 (0.951-1.030)	0.626 (0.595-0.689)	0.908 (0.872-0.956)

Supplementary Table S6: Map similarity between ground-truth maps and estimated spatial maps associated with k-means, PCA, and ICA (note HMM states are defined in terms of (lagged) covariance matrices and not spatial maps, so HMM is excluded from this table). Values are Fisher z-scored for better differentiation between high values, and shown are median and interquartile range (in brackets). Abbreviations as in Table S5

.

such as bursts will likely have unique spatial patterns, which again will reflect as patterns of activation. Furthermore, in our study we found a significant association between patterns of activation and functional connectivity, suggesting that patterns of activation likely drive covariance matrices. A detailed study of how source-space estimates of brain states from different algorithms in real data would be of much interest.

References

Abeysuriya R.G., Hadida J., Sotiropoulos S.N., Jbabdi S., Becker R., Hunt B.A.E., Brookes M.J., and Woolrich M.W. A biophysical model of dynamic balancing of excitation and inhibition in fast oscillatory large-scale networks. *PLoS Comput Biol*, 14(2):e1006007, 2018. doi: 10.1371/journal.pcbi.1006007.

Arthur D. and Vassilvitskii S. K-means++: The advantages of careful seeding. In *Discrete Algorithms: SODA-07 Proceedings of the Eighteenth Annual ACM-SIAM Symposium*, page 1027–1035, 2007.

Baker A.P., Brookes M.J., Smith S.M., Behrens T., Probert Smith P.J., and Woolrich M. Fast transient networks in spontaneous human brain activity. *eLife*, 3:e01867, 2014. doi: 10.7554/eLife.01867.

Colclough G.L., Woolrich M.W., Tewarie P.K., Brookes M.J., Quinn A.J., and Smith S.M. How reliable are MEG resting-state connectivity metrics? *Neuroimage*, 138:284–293, 2016. doi: 10.1016/j.neuroimage.2016.05.070.

Custo A., Van De Ville D., Wells W.M., Tomescu M.I., Brunet D., and Michel C.M. Electroencephalographic resting-state networks: Source localization of microstates. *Brain Conn*, 7(10):671–682, 2017. doi: 10.1089/brain.2016.0476.

Deco G., Jirsa V., McIntosh A.R., Sporns O., and Kötter R. Key role of coupling, delay, and noise in resting brain fluctuations. *Proc Natl Acad Sci USA*, 106(25):10302–10307, 2009. doi: 10.1073/pnas.0901831106.

Hatz F., Hardmeier M., Benz N., Ehrensperger M., Gschwandtner U., Rüegg S., Schindler C., Monsch A.U., and Fuhr P. Microstate connectivity alterations in patients with early Alzheimer's disease. *Alz Res Therapy*, 7:78, 2015. doi: 10.1186/s13195-015-0163-9.

Hyvarinen A. Fast and robust fixed-point algorithms for independent component analysis. *IEEE Trans Neural Net*, 10(3): 626–634, 1999. doi: 10.1109/72.761722.

Khanna A., Pascual-Leone A., and Farzan F. Reliability of resting-state microstate features in electroencephalography. *PLoS One*, 9(12):e114163, 2014. doi: 10.1371/journal.pone.0114163.

Khanna A., Pascual-Leone A., Michel C.M., and Farzan F. Microstates in resting-state EEG: Current status and future directions. *Neurosci Biobehav Rev*, 49:105–113, 2015. doi: 10.1016/j.neubiorev.2014.12.010.

Koenig T. and Brandeis D. Inappropriate assumptions about EEG state changes and their impact on the quantification of EEG state dynamics. *Neuroimage*, 125:1104–1106, 2016. doi: 10.1016/j.neuroimage.2015.06.035.

Koenig T., Lehmann D., Merlo M.C.G., Kochi K., D. Hell, and M. Koukkou. A deviant EEG brain microstate in acute, neuroleptic-naive schizophrenics at rest. *Eur Arch Psychiatry Clin Neurosci*, 249:205–211, 1999. doi: 10.1007/s004060050088.

Michel C.M. and Koenig T. EEG microstates as a tool for studying the temporal dynamics of whole-brain neuronal networks: A review. *NeuroImage*, 180(B):577–593, 2018. doi: 10.1016/j.neuroimage.2017.11.062.

Michel C.M., Koenig T., and Brandeis D. Electrical neuroimaging in the time domain. In Michel C.M., Koenig T., Brandeis D., Gianotti L.R.R., and Wackermann J., editors, *Electrical Neuroimaging*, pages 111–143. Cambridge University Press, 2009.

Murray M.M., Brunet D., and Michel C.M. Topographic ERP analyses: A step-by-step tutorial review. *Brain Topogr*, 20: 249–264, 2008. doi: 10.1007/s10548-008-0054-5.

O'Neill G.C., Tewarie P., Vidaurre D., Liuzzi L., Woolrich M.W., and Brookes M.J. Dynamics of large-scale electrophysiological networks: A technical review. *Neuroimage*, 180(B):559–576, 2018. doi: 10.1016/j.neuroimage.2017.10.003.

Pascual-Marqui R.D., Michel C.M., and Lehmann D. Segmentation of brain electrical activity into microstates: model estimation and validation. *IEEE Trans Biomed Eng*, 42(7):658–665, 1995. doi: 10.1109/10.391164.

Rousseeuw P.J. Silhouettes: A graphical aid to the interpretation and validation of cluster analysis. *J Comput Appl Math*, 20:53–65, 1987. doi: 10.1016/0377-0427(87)90125-7.

Rummel C., Abela E., Müller M., Hauf M., Scheidegger O., Wiest R., and Schindler K. Uniform approach to linear and non-linear interrelation patterns in multivariate time series. *Phys Rev E*, 83:066215, 2011. doi: 10.1103/PhysRevE.83.066215.

Rummel C., Abela E., Andrzejak R.G., Hauf M., Pollo C., Müller M., Weisstanner C., Wiest R., and Schindler K. Resected brain tissue, seizure onset zone and quantitative EEG measures: Towards prediction of post-surgical seizure control. *PLoS One*, 10(10):e0141023, 2015. doi: 10.1371/journal.pone.0141023.

Satopää V., Albrecht J., Irwin D., and Raghavan B. Finding a "kneedle" in a haystack: Detecting knee points in system behavior. In *2011 31st International Conference on Distributed Computing Systems Workshops*, pages 166–171, 2011. doi: 10.1109/ICDCSW.2011.20.

Schreiber T. and Schmitz A. Surrogate time series. *Physica D*, 142(3-4):346–382, 2000. doi: 10.1016/S0167-2789(00)00043-9.

Smith S.M., Fox P.T., Miller K.L., Glahn D.C., Fox P.M., Mackay C.E., Filippini N., Watkins K.E., Toro R., Laird A.R., and Beckmann C.F. Correspondence of the brain's functional architecture during activation and rest. *Proc Natl Acad Sci USA*, 106(31):13040–13045, 2009. doi: 10.1073/pnas.0905267106.

Tait L., Tamagnini F., Stothart G., Barvas E., Monaldini C., Frusciante R., Volpini M., Guttmann S., Coulthard E., Brown J.T., Kazanina N., and Goodfellow M. EEG microstate complexity for aiding early diagnosis of Alzheimer's disease. *Sci Rep*, 10:17627, 2020. doi: 10.1038/s41598-020-74790-7.

Treder M.S. MVPA-light: A classification and regression toolbox for multi-dimensional data. *Front Neurosci*, 14:289, 2020. doi: 10.3389/fnins.2020.00289.

Van De Ville D., Britz J., and Michel C.M. EEG microstate sequences in healthy humans at rest reveal scale-free dynamics. *Proc Natl Acad Sci USA*, 107(42):18179–18184, 2010. doi: 10.1073/pnas.1007841107.

Vidaurre D., Hunt L.T., Quinn A.J., Hunt B.A.E., Brooked M.J., Nobre A.C., and Woolrich M.W. Spontaneous cortical activity transiently organises into frequency specific phase-coupling networks. *Nat Commun*, 9:2987, 2018. doi: 10.1038/s41467-018-05316-z.

Vinck M., Oostenveld R., van Wingerden M., Battaglia F., and Pennartz C.M.A. An improved index of phase-synchronization for electrophysiological data in the presence of volume-conduction, noise and sample-size bias. *Neuroimage*, 55(4): 1548–1565, 2011. doi: 10.1016/j.neuroimage.2011.01.055.

von Wegner F., Tagliazucchi E., Brodbeck V., and Laufs H. Analytical and empirical fluctuation functions of the EEG microstate random walk - short-range vs. long-range correlations. *Neuroimage*, 141:442–451, 2016. doi: 10.1016/j.neuroimage.2016.07.050.

von Wegner F., Tagliazucchi E., and Laufs H. Information-theoretical analysis of resting state EEG microstate sequences - non-markovianity, non-stationarity and periodicities. *Neuroimage*, 158:99–111, 2017. doi: 10.1016/j.neuroimage.2017.06.062.

von Wegner F., Knaut P., and Laufs H. EEG microstate sequences from different clustering algorithms are information-theoretically invariant. *Front Comput Neurosci*, 12:70, 2018. doi: 10.3389/fncom.2018.00070.

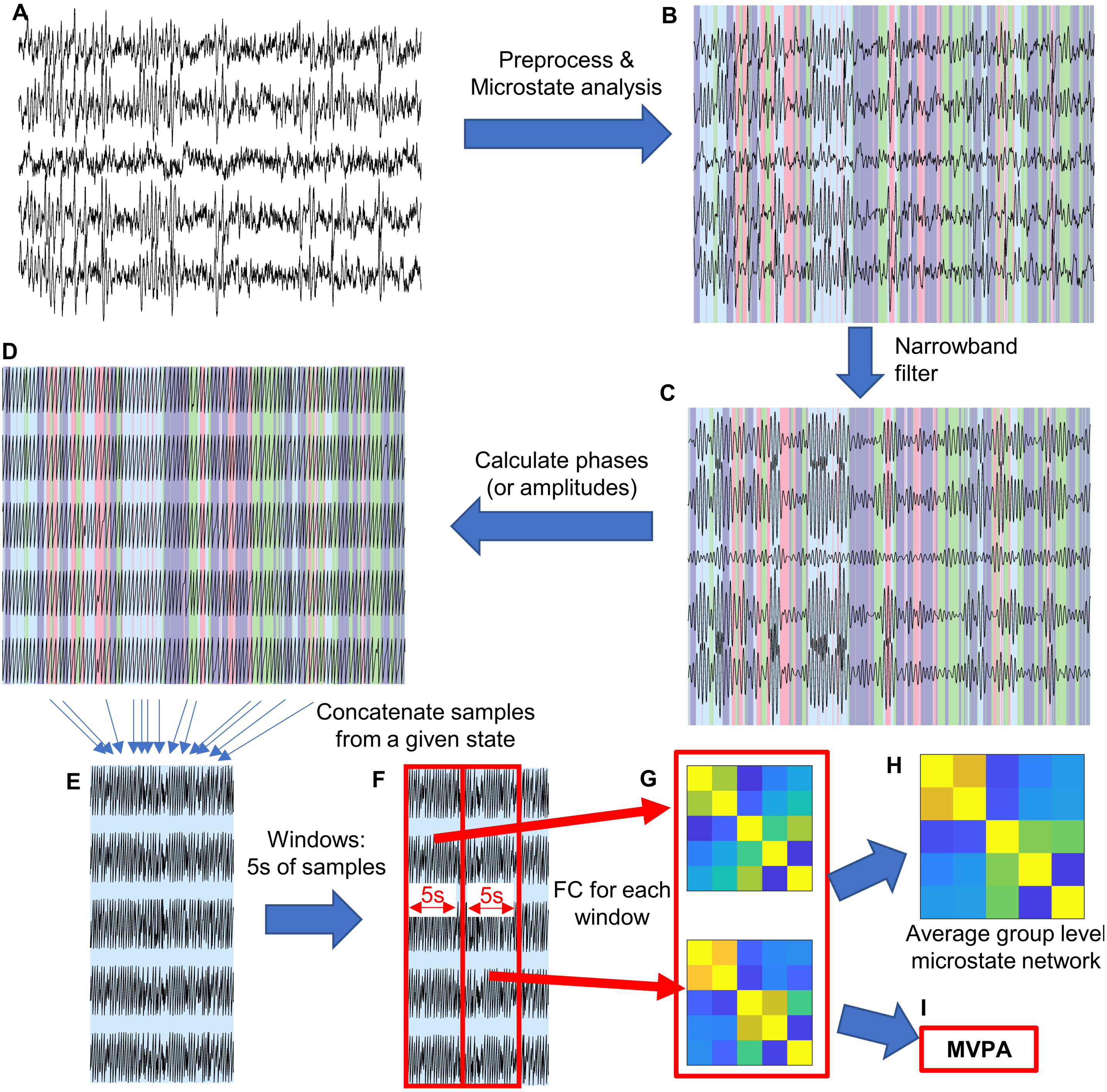

Supplementary Figure S1: **Calculating microstate functional connectivity**. (A) Raw MEG source space data. Note the data shown here is simulated data for visualization purposes, but empirical data was used in the analysis. (B) By appropriately preprocessing and performing microstate analysis on the data, we can assign each sample a microstate class (denoted here by colour). (C) To calculate narrowband functional connectivity, the data must be filtered into an appropriate frequency band. (D) Using the Hilbert transform, calculate instantaneous phases or amplitudes from the narrowband data. (E) Concatenate all samples with a label according to a given state. Here, we have shown all samples from the state with a light-blue background. (F) Window the concatenated data into epochs with 5 seconds worth of samples. (G) For each window, calculate functional connectivity. Here, we used wPLI. (H) To obtain a group level representation of the functional network associated with this microstate, average all functional connectivity matrices derived from this state. (I) To statistically test whether microstates are associated with a specific functional network structure, each matrix (along with the sets of matrices from all other microstate classes) can be submitted for multivariate pattern analysis.

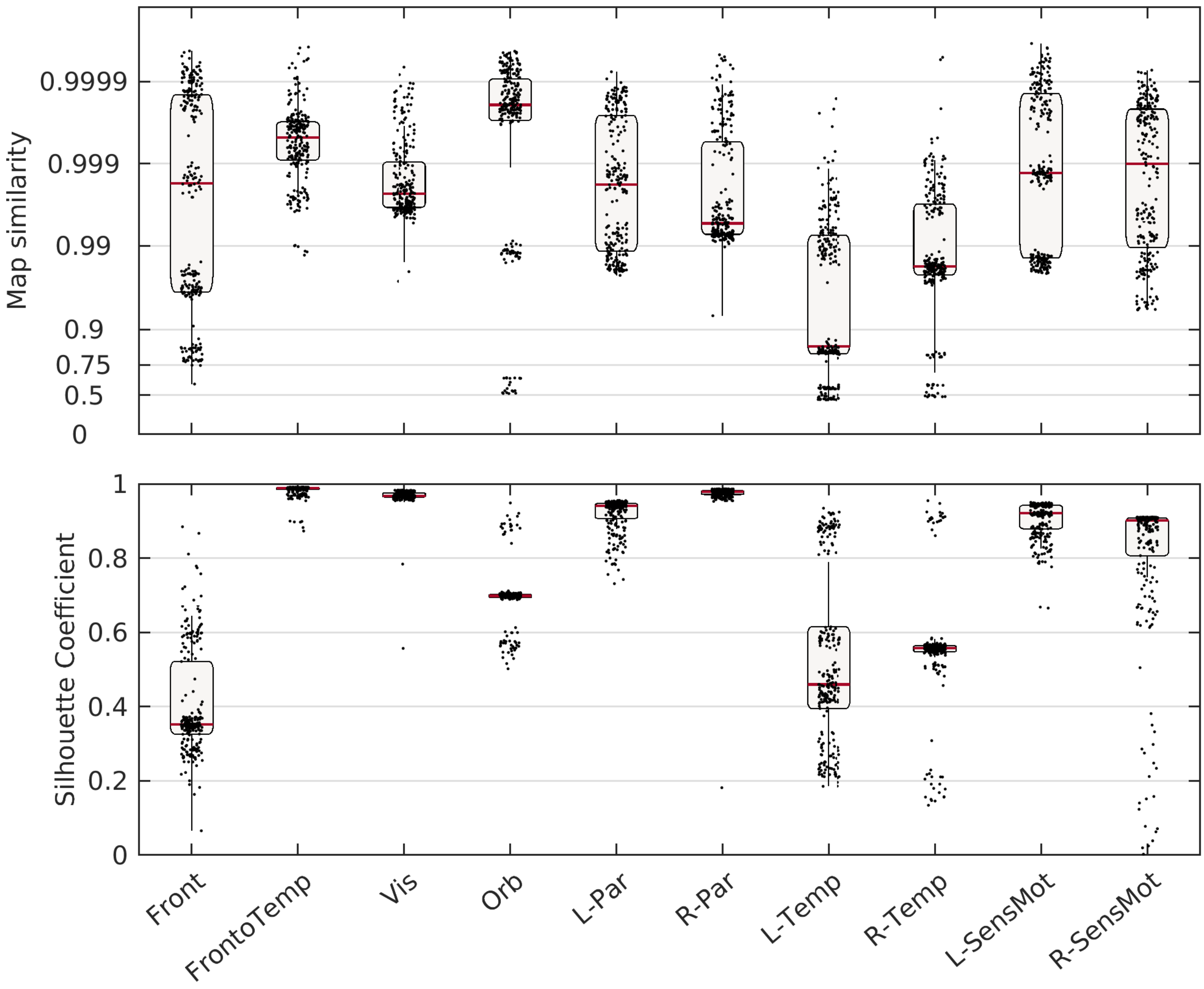

Supplementary Figure S2: **Reproducibility of resting-state microstates**. The k-means clustering algorithm was repeated 250 times with different sets of GFP peaks and initial seeds, and then matched to the original set of maps using a template matching algorithm. Each point correponds to a map estimated by a different repeat of the algorithm, while the classification according to the original maps is given by the x-axis. (Top) Map similarity between the estimated and original maps. The y-axis labels correspond to the raw values of map similarity, but the axis itself was transformed according to a Fisher z-transform to help differentiate large values of map similarity. (Bottom) Silhouette coefficients of each point.

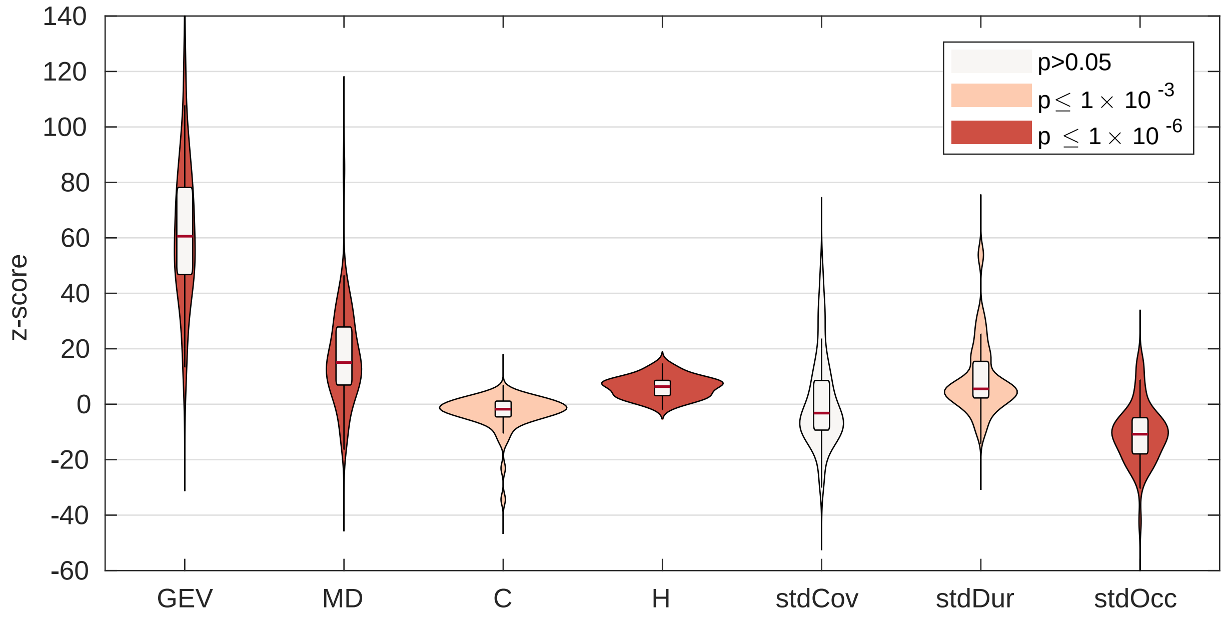

Supplementary Figure S3: **z-scores of empirical microstate statistics vs stationary surrogate distributions.**

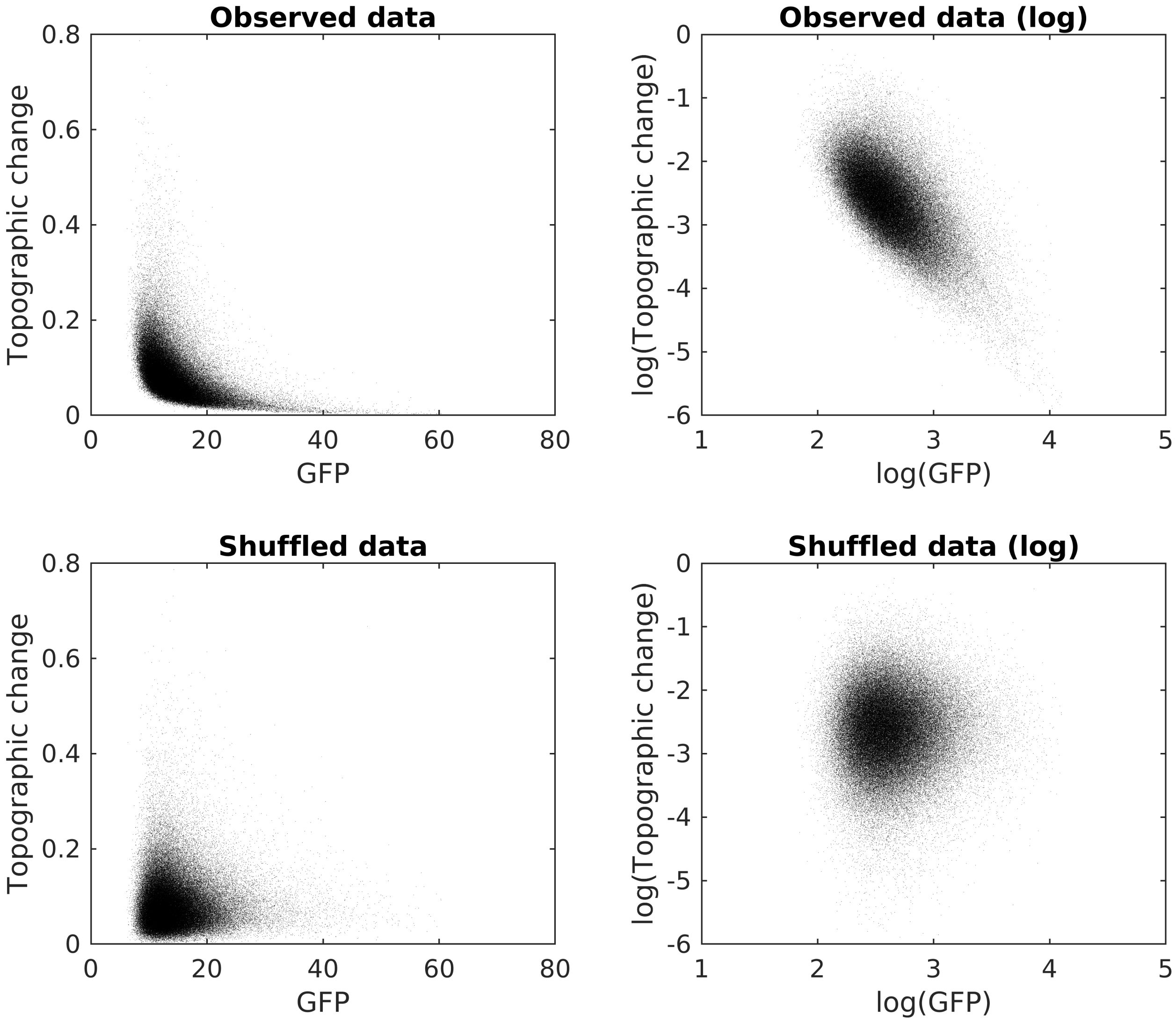

Supplementary Figure S4: **GFP vs topographic change.** Here, we recreate Fig 1 from Koenig and Brandeis (2016) using resting-state source MEG microstates, with modified definitions of GFP and map (dis)similarity as described in section S1.1. Upper rows show the observed data, while lower rows show shuffled data to simulate assumptions of Hidden Markov Modelling (i.e. independence of GFP and topographic change; Koenig and Brandeis (2016)). Left column shows original data, while right column shows log-log transformed data. After log-log transformation, there is a correlation of -0.6225 ± 0.0189 (mean $\pm$ standard error across participants). The data shown here is from a single representative participant, with correlation of -0.6466. This figure demonstrates that the inverse relationship between GFP and the topographic change holds in MEG source space data, justifying the use of GFP peaks (as the most stable samples) in the k-means clustering algorithm and demonstrating that the assumptions of Hidden Markov Modelling may be inappropriate for this data (Koenig and Brandeis, 2016).

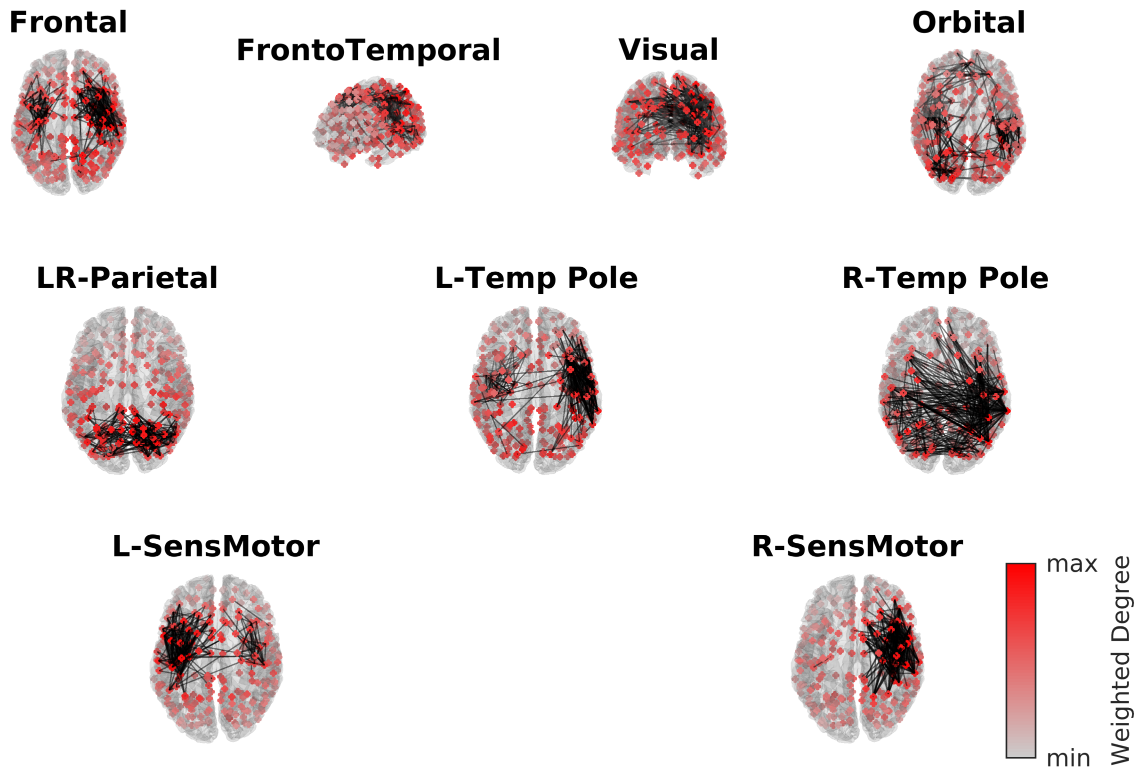

Supplementary Figure S5: **Connectivity results for auditory paradigm.**
